# Longitudinal Trend Evaluation and Prescription Cost Analysis (PCA) of Clozapine in the United Kingdom

**DOI:** 10.1192/bjo.2024.150

**Published:** 2024-08-01

**Authors:** Nadeem Gire, Atta Asif, Nusrat Husain

**Affiliations:** 1 University of Bolton, Bolton, United Kingdom; 2 Ethnic Health Forum, Manchester, United Kingdom; 3 University of Manchester, Manchester, United Kingdom

## Abstract

**Aims:**

Severe Mental Illnesses (SMI) are a group of disorders which can have a debilitating impact on an individual's daily life functioning. The National Institute for Health and Care Excellence (NICE) has set out clinical guidelines for the treatment of SMI including the use of Second Generation Antipsychotic (SGA) medication as well as psychological therapies. However, Treatment Resistant Schizophrenia (TRS) affects approximately 34% of patients with schizophrenia. Clozapine, a SGA, has shown superiority in treatment resistant schizophrenia as well as its potential benefits in reducing suicidality and improving functioning.

**Methods:**

The following study aimed to examine the longitudinal trends in prescribing clozapine based on the NHS Digital prescription cost analysis (PCA) between 2015–2023.

**Results:**

The results show that a number of prescriptions decrease simultaneously from the financial year 2015 (n = 5536) to 2023 (n = 3059). The cost was also found to be reducing until the financial year 2018–19 where there was an increase in costs which reached the maximum (14%) despite the number of prescriptions being lower as compared with 2015–16. In addition, it was found that clozapine prescribing trends have been reducing over time, despite a large proportion of service users with schizophrenia experiencing TRS (34%). Overall, since 2015–2023 a total of n = 34,440 items of clozapine were prescribed costing £1,252,052.27.

**Conclusion:**

Considering clozapine's superior efficacy in the treatment of TRS, further research is required to better understand prescribing practices, monitoring compliance of clozapine and treatment adherence. Further qualitative research is needed to better understand the views and perspectives of both service users and prescribers in the clinical use of clozapine. Future research may also look at referrals of clozapine-prescribed patients to psychological services, the impact of clozapine in TRS patients who are offered psychological therapy, and the potential clinical and cost implications of not prescribing clozapine.

